# Supplementary material for: The Geometry of Layer 2/3 Cortical Sound Processing in Slow Wave Sleep
Source: Adv Sci (Weinh). 2025 Nov 18;13(7):e09707. doi: 10.1002/advs.202509707 (PMC12866753; doi:10.1002/advs.202509707)
Supplement: Supplementary file 1 — Supporting Information [file ADVS-13-e09707-s001.pdf]

Supporting Information

The Geometry of Layer 2/3 Cortical Sound Processing in Slow Wave Sleep

*Allan Muller, Anton Filipchuk, Sophie Bagur, Brice Bathellier**

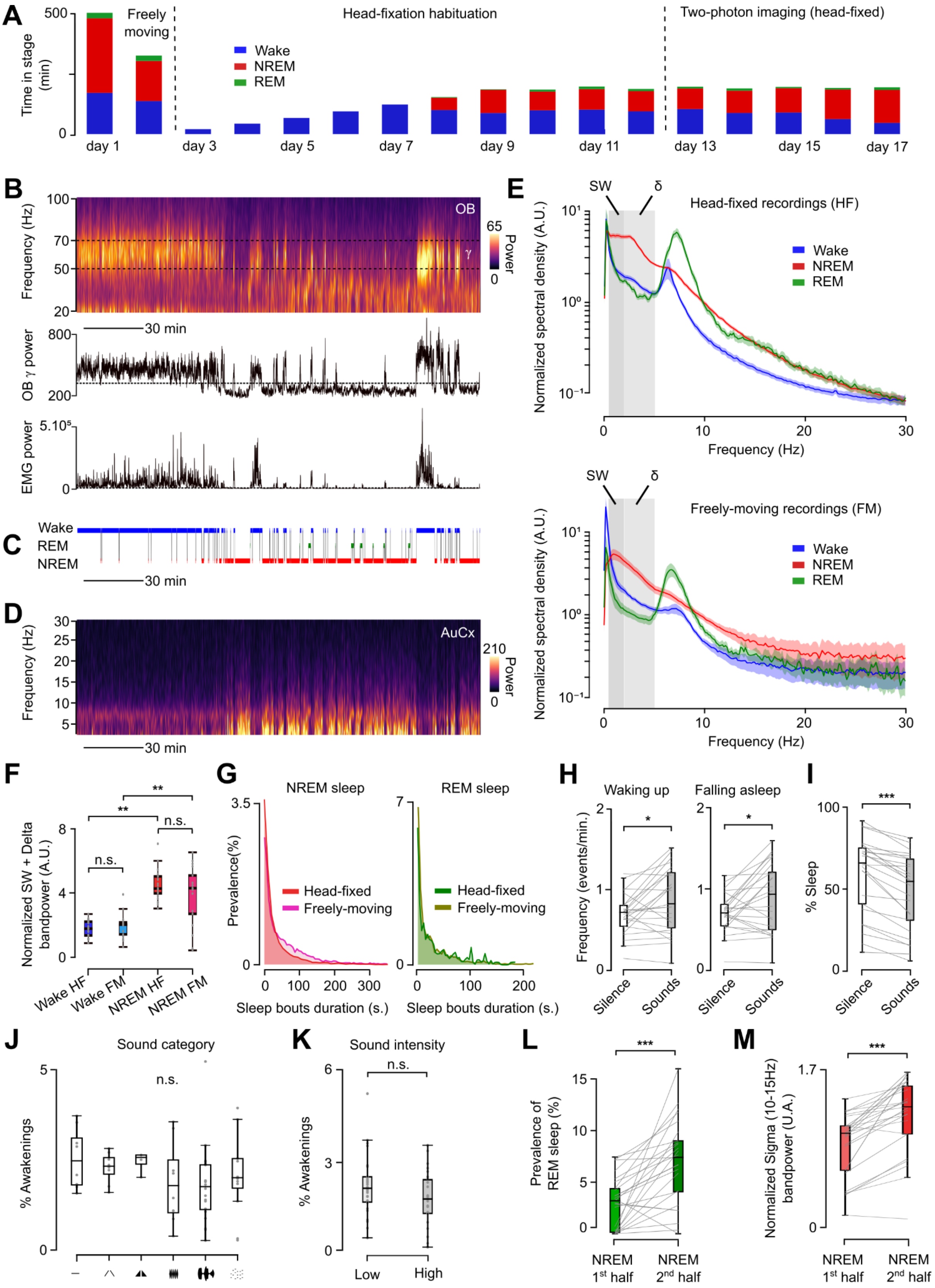

Figure S1: **OB gamma and EMG power identify sleep periods in head-fixed mice. A,** Time spent in Wake, NREM and REM states during training and imaging sessions for a representative mouse head-fixed without a running-wheel (box condition). **B,** Spectrogram of Olfactory Bulb LFP throughout an entire recording session (top). The evolution of the gamma bandpower (middle) follows EMG power (bottom), and similarly distinguishes sleep, when it is low, from wakefulness, when it is high. **C,**

Corresponding hypnogram. **D,** Corresponding auditory cortex spectrogram showing increases in low-frequency power during sleep periods. **E,** Power spectra of auditory cortex LFP during wakefulness, NREM, and REM sleep periods computed with Welch method show similar frequency density when mice were head-fixed (top) or in freely-moving condition (bottom). Power was divided by averaged spectrogram value to allow comparison between recordings. Grey area shows the slow-wave (0.5-2 Hz) and Delta (2-5 Hz) frequency bands. **F,** Low frequency (0.5-5 Hz) power is comparable between head-fixed (HF, n=25 recordings) and freely-moving (FM, n=16 recordings) conditions during wake (Mann-Whitney U test,: $p=8.6x10^{-1}$) and NREM sleep (Mann-Whitney U test, $p=5.4x10^{-1}$) and systematically increases in NREM sleep compared to wakefulness (Mann-Whitney U test, HF: $p=1.4x10^{-9}$; FM: $p=4.7x10^{-3}$). **G,** Distribution of sleep bouts duration shows a higher proportion of short NREM sleep periods in head-fixed mice compared to freely-moving but similar REM sleep durations. **H,** Number of wake-up (left) and fall-asleep (right) events slightly increase in "sounds" blocks compared to "blank" blocks (Wilcoxon signed rank test, wake-up: $p=2.0x10^{-2}$; fall-asleep: $p=3.4x10^{-2}$, n=25 recordings). **I,** Percentage of time spent asleep slightly decreases in "sounds" blocks compared to "blank" blocks (Wilcoxon signed rank test, $p=2.5x10^{-3}$, n=25 recordings). **J** Fraction of sound presentations resulting in the mouse awakening for 6 different sound categories (pure tone, FM sweeps, intensity ramps, AM sounds, complex sounds, white and colored noises). No significant difference across categories is observed (Kruskal-Wallis test, p=0.16). **K,** same as J but for lower (50-60 dB SPL) and higher (70-80 dB SPL) loudness sounds (Mann-Whitney U test, p=0.11). **L,** the fraction of time spent in REM sleep (Wilcoxon signed rank $p=2.7 \times 10^{-5}$) and **M,** the sigma band power is larger during the second half of sleep in the imaging sessions (Wilcoxon signed rank, $p=3.0x10^{-7}$, n=24 recordings)

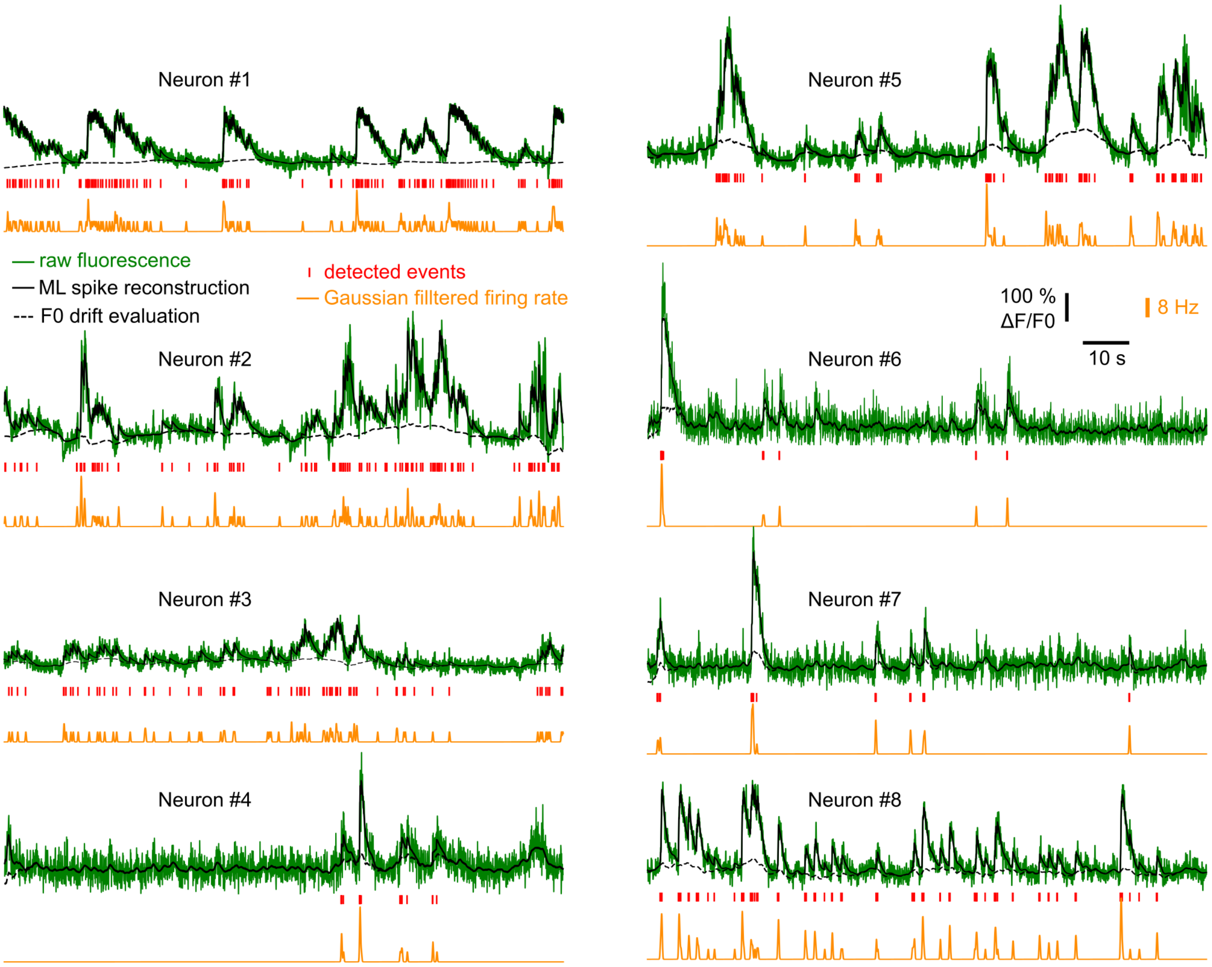

Figure S2: **Sample fluorescence traces and ML Spike deconvolution.** For 8 different sample neurons: raw fluorescence traces (green line) and individual events detected by the MLSpike algorithm (red bars). The reconstruction of the fluorescence by MLSpike (black line) sums individual transients detected by the algorithm (red) and the drift evaluation trace (black dashed line). The instantaneous firing rate is evaluated by applying a Gaussian smoothing to the detected transients (standard deviation 0.1 s, orange line).

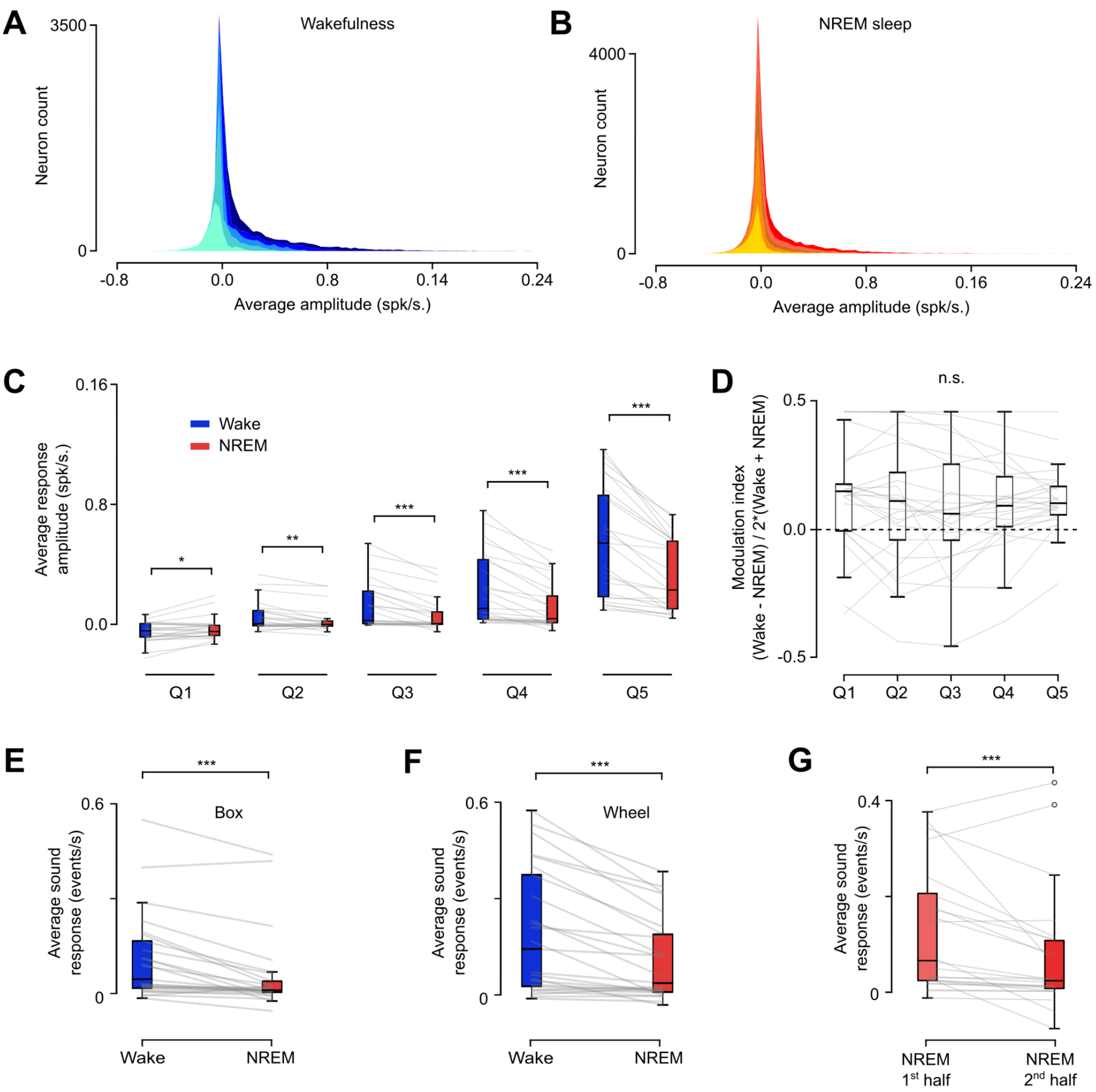

Figure S3: **Comparison of response firing rates in different states. A,** Distribution of average neuronal responses to individual sounds across all neurons during wakefulness, illustrating a highly skewed distribution. The average neural responses are subtracted by the average spontaneous activity measured in a 0.5 s baseline time bin before sound onset. Neurons are stratified into quintiles based on their average firing activity across all sounds in the sessions during wakefulness, with colors ranging from lightest (lowest 20th percentile) to darkest (highest 20th percentile). **B,** Same as **A** during NREM sleep. **C,** Mean sound-evoked response relative to baseline for each neuronal firing rate quintile during Wake and NREM sleep states, demonstrating a systematic reduction in response magnitude during sleep across all quintiles (Wilcoxon signed rank test, "Q1": $p=2.4x10^{-2}$, "Q2": $p=5.1x10^{-3}$, "Q3": $p=8.3x10^{-7}$, "Q4": $p=6.0x10^{-8}$, "Q5": $p=1.2x10^{-7}$, n=25 recordings) . **D,** Modulation index quantifying the change in sound-evoked activity between NREM and Wake states for each quintile, showing no significant differences across groups (Friedman test, $p=7.3x10^{-2}$, n=25 recordings). **E-F,** Averaged sound response amplitude of neurons decreases in NREM sleep either when the animal was trained to sleep in a box (no locomotion possible, Wilcoxon signed rank test, $p=5.2x10^{-6}$, n=13 recordings) or on a wheel (locomotion possible, Wilcoxon signed rank test, $p=5.4x10^{-5}$, n=12 recordings). **G,** Averaged sound

response amplitude decreased in the second half of NREM sleep in the imaging sessions (Wilcoxon signed rank test, p= 4.3×10^{-4}, n=24 recordings).

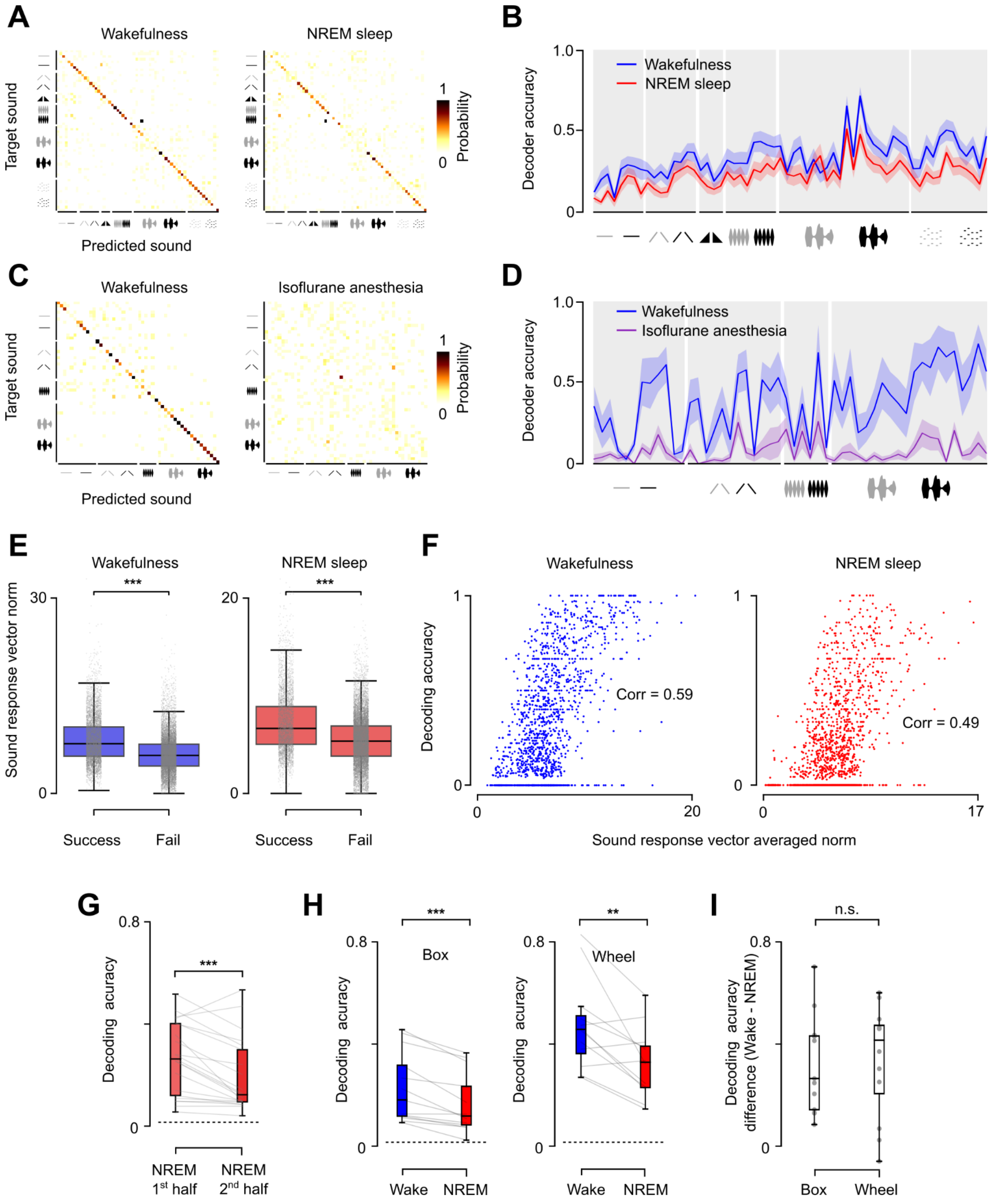

Figure S4: **Comparison of sound decoding in different states. A,** Confusion matrices recapitulating classification outcomes of sound decoders from wakefulness (left) and NREM sleep (right) neuronal activity. **B,** Decoding accuracy for every sound stimulus in wakefulness and NREM sleep (chance level is 1.67%). **C,** Confusion matrices recapitulating classification outcomes of sound decoders from wakefulness (left) and isoflurane anesthesia (right) neuronal activity. **D,** Decoding accuracy for every sound stimulus in wakefulness and isoflurane induced anesthesia (change level is 2%). **E,** Population response is stronger in trials where the decoder succeeded in classifying sound stimuli both in wakefulness (left, Mann-Whitney U test, p = 0.0) and NREM sleep (right, Mann-Whitney U test, $p=7.3 \times 10^{-241}$). **F,** Decoding accuracy of a sound positively correlates with the averaged response

intensity of that sound in both wakefulness ($p = 2.6x10^{-141}$) and NREM sleep. ($p = 8.6x10^{-89}$). **G,** Sound identity decoding accuracy during NREM in the first half (lighter sleep) and in the second half (deeper sleep) of the recording sessions (Wilcoxon signed rank test, $p=5.1x10^{-4}$, n=24 recordings, chance level is 1.67% - dashed line). **H,** Sound decoding accuracy in wake and NREM sleep for mice trained to sleep in a box (no locomotion possible, left, Wilcoxon signed rank test, $p=2.4x10^{-4}$, n=13 recordings) or on a wheel (locomotion possible, right, Wilcoxon signed rank test, $p=2.4x10^{-3}$, n=12 recordings). **I,** Difference of decoding accuracy between wake and NREM sleep for mice trained in the box or on the wheel (Mann-Whitney U test, $p=6.1x10^{-1}$, n=12 and 13 recordings).

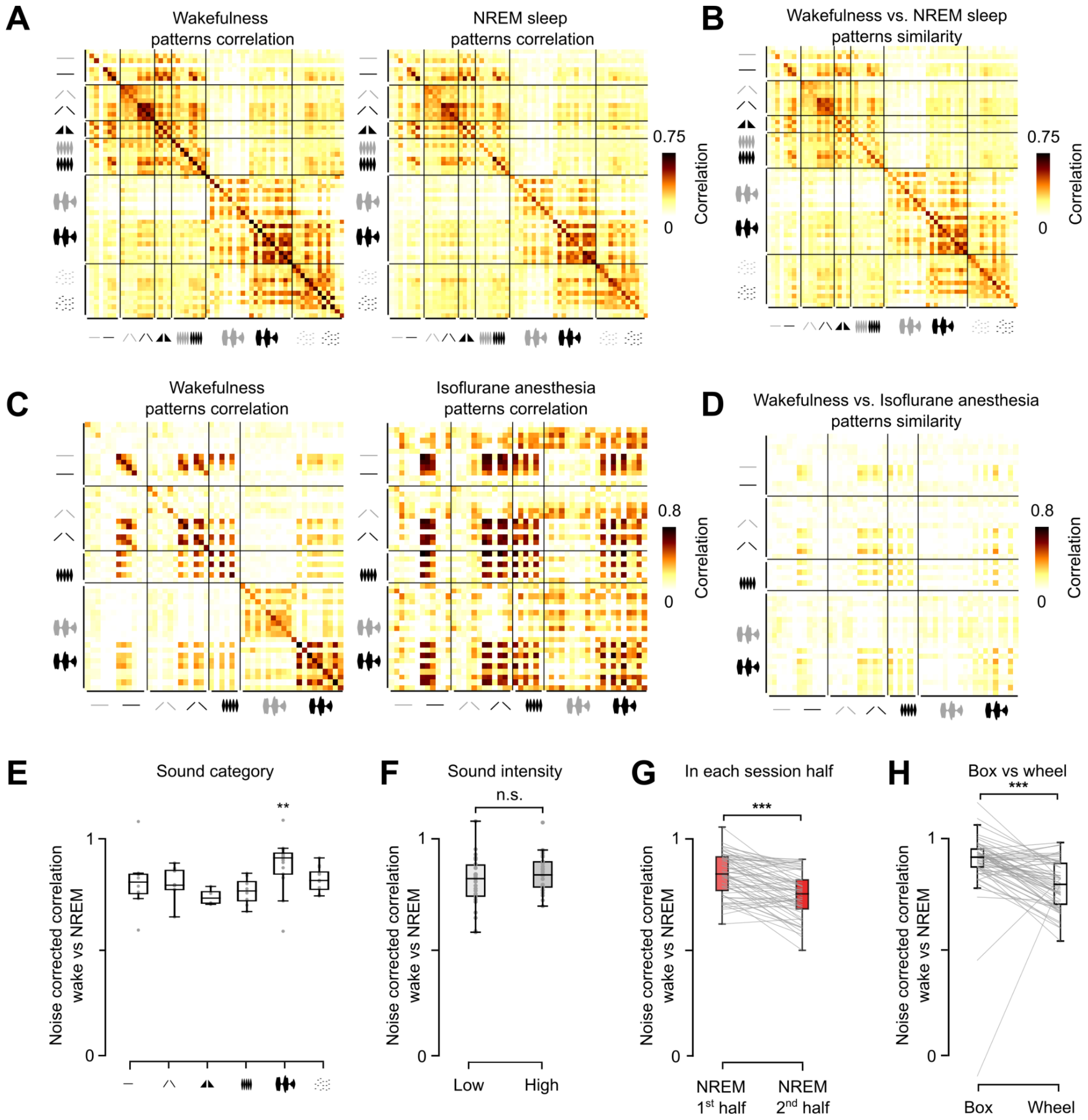

Figure S5: **Within-state and cross-state representation similarity analysis: NREM sleep vs wake and anesthesia vs wake. A,** Non noise corrected similarity matrices between population sound-evoked responses averaged over two distinct halves of the sound-presentations in wakefulness (left), and in NREM sleep (right). **B,** Non noise corrected similarity matrices between sound-evoked responses in wakefulness and NREM sleep. **C,** Same as **A** but for wakefulness and isoflurane anesthesia. **D,** Same as **B** for wakefulness and isoflurane anesthesia. **E,** Mean noise-corrected correlation between NREM and wake averaged over 6 different sound categories (pure tone, FM sweeps, intensity ramps, AM sounds, complex sounds, white and colored noises). Sound representations are slightly more similar between NREM and wake for complex sounds (Kruskal-Wallis test, $p=1.1\times10^{-3}$). **F,** Same as E but for lower (50-60 dB SPL) and higher (70-80 dB SPL) intensity sounds (Mann-Whitney U test, p=0.25). **G,** Noise corrected correlation between representations of the same sounds in wake and in NREM sleep is larger for the first half than for the the second half of the sessions (Wilcoxon signed rank test, $p=4.1\times10^{-8}$, n=60 sounds). **H,** Noise corrected correlation between representations of the same sounds in wake

and in NREM sleep is larger when mice are head fixed in a box than when that are head-fixed on a wheel and can locomote in wakefulness (Wilcoxon signed rank test, $p=5.0x10^{-7}$, n=60 sounds).

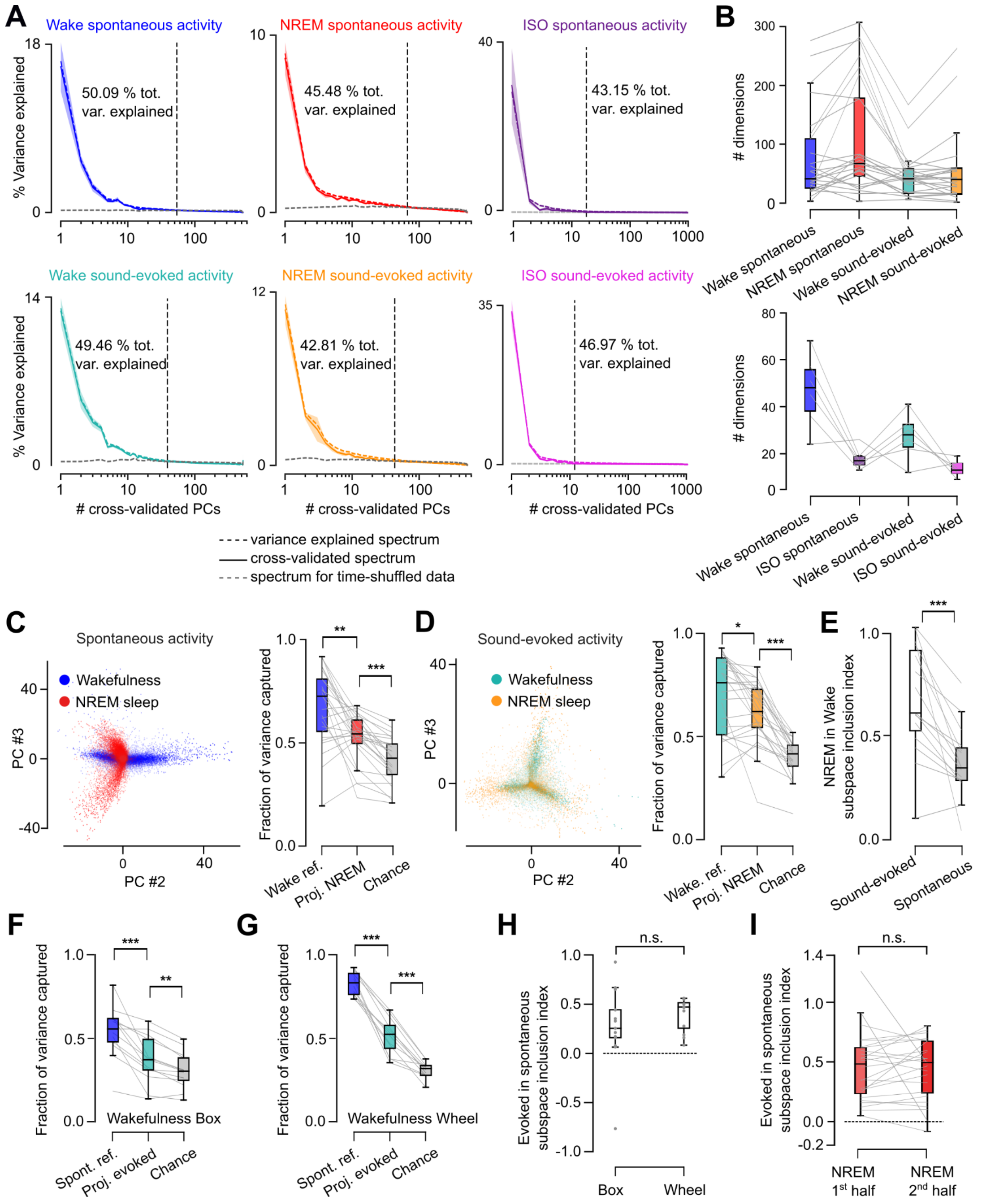

Figure S6: Geometry of spontaneous and evoked activity across sleep and wake. A, Scree plots from PCA (dashed) and cross-validated PCA (solid line, see methods) performed on wake, NREM, and isoflurane anesthesia, spontaneous and sound-evoked neuronal activity. Note that the two curves strongly overlap. The number of dimensions used to describe each subspace was taken as the number of principal components (coloured) that capture more variance than expected by chance (grey), and is here denoted as a vertical grey dashed line. About 50% of total variance is usually captured in that subspace. **B,** Number of components used to describe each condition subspace. **C,** Left: 2D example projection of wakefulness and NREM sleep spontaneous neuronal activity in principal component (PC) space. Right: Fraction of variance of spontaneous wakefulness and NREM sleep neuronal activity

explained by dimensions of the spontaneous wakefulness subspace (Wilcoxon signed rank test, "Wake reference" to "Projected NREM": $p=2.5x10^{-3}$; "Projected NREM" to "chance level": $p=6.0x10^{-8}$, n=25). **D,** Same as **C** for sound-evoked activity (Wilcoxon signed rank test, "Wake reference" to "Projected NREM": $p=2.7x10^{-2}$; "Projected NREM" to "chance level": $p=6.0x10^{-8}$, n=25). **E,** The inclusion index of NREM sleep sound-evoked activity in wake sound-evoked subspace is higher than the inclusion index of spontaneous NREM sleep spontaneous activity in wake spontaneous subspace (Wilcoxon signed rank test, $p=1.2x10^{-4}$, n=15). Sessions where the maximum overlap expected value did not exceed chance level by 0.1 were removed. **F.** Fraction of variance of spontaneous wakefulness and NREM sleep neuronal activity explained by dimensions of the spontaneous wakefulness subspace (Wilcoxon signed rank test, "Spontaneous reference" to "Projected evoked": $p=2.4x10^{-4}$; "Projected evoked" to "chance level": $p=1.7x10^{-3}$, n=25 recordings). **G,** Same as **F** when the animal was recorded on the wheel (Wilcoxon signed rank test, "Spontaneous reference" to "Projected evoked": $p=4.9x10^{-4}$; "Projected evoked" to "chance level": $p=4.9x10^{-4}$, n=25 recordings). **H,** Subspace inclusion indices during wakefulness quantifying the level of inclusion of sound-evoked activity into spontaneous subspace showed no differences in animals trained to sleep in a box (no locomotion) or on a wheel (locomotion allowed) (Mann-Whitney U test, $p=5.3x10^{-2}$, n=13 box recordings and n=12 wheel recordings). **I,** Subspace inclusion index did not vary from the first to the second half of NREM sleep in the imaging sessions (Wilcoxon signed rank test, $p= 4.2x10^{-1}$, n=24 recordings).

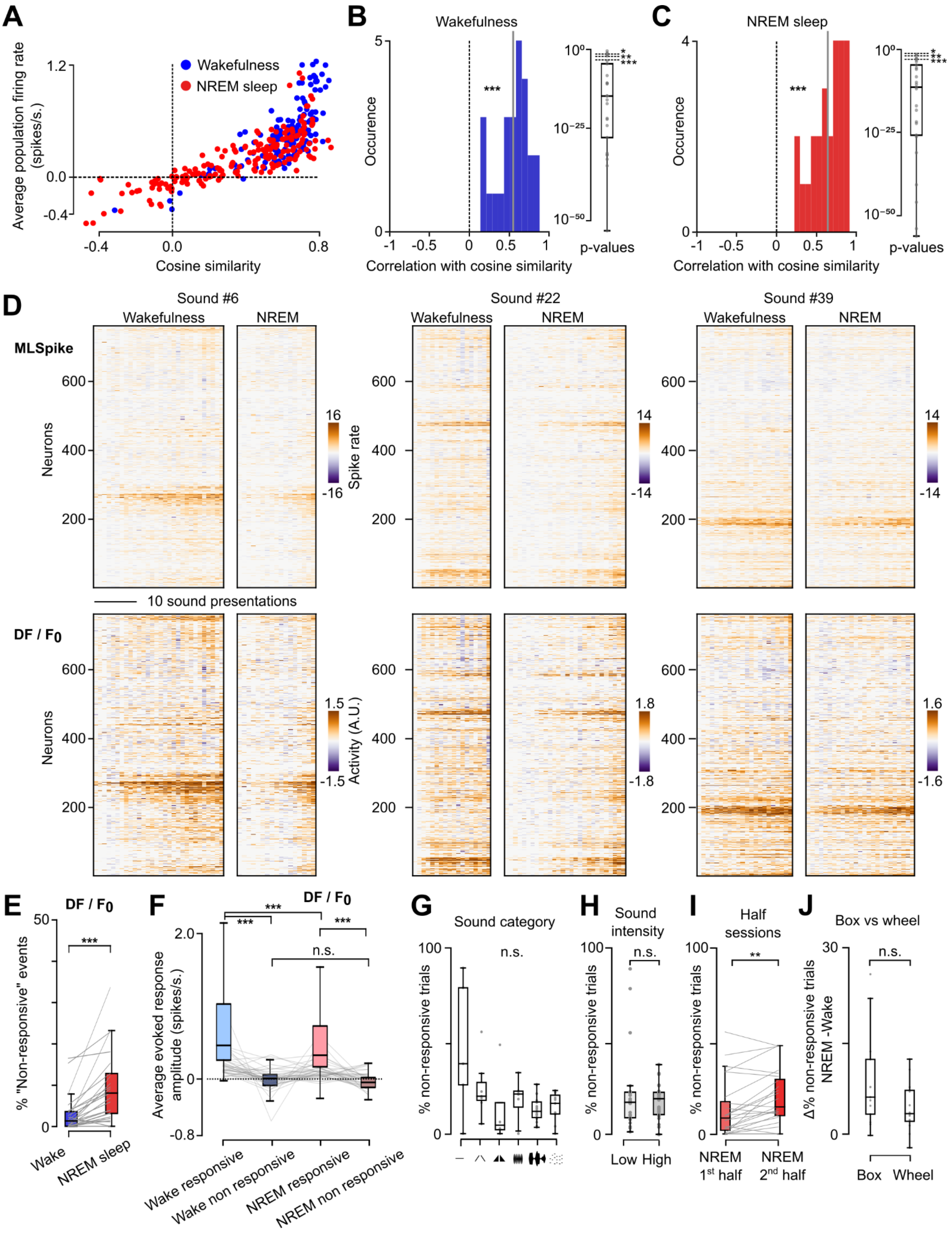

Figure S7: Sound encoding reduction is explained by population response gain, and increases with Delta and Sigma oscillatory power in NREM sleep but not in wake. A, Example of positive relationship between amplitude of population responses to sounds and their similarity to the expected response vector in wakefulness and NREM sleep from a single recording. **B,** Left: distribution of Pearson correlation coefficients between the amplitude of population responses to sounds and their similarity to the expected response vector in wakefulness for every recording (Wilcoxon signed rank rank test, p=6.0x10^{-8}, n=25). The solid grey vertical line indicates the mean of the distribution. Right:

correlation p-values. Dashed lines indicate common values to assess statistical significance (* = 0.05, ** = 0.01, *** = 0.001). **C,** Same as **B** in NREM sleep (left, Wilcoxon signed rank test, p=6.0,10^{-8}, n=25 recordings). **D,** Population response (mean response amplitude of each neuron and sound presentation color-coded) for a sample population and three sample sounds. The activity (response window - baseline) is measured either based on MLSpike events (top) or based on the raw ΔF/F signal (bottom). **E,** Fraction of non responsive trials, computed based on the raw ΔF/F signal instead of ML Spike events (Wilcoxon signed rank test, p=3.9×10^{-5}, n=24 recordings). **F,** Average population response for responsive and non-responsive trial in wake and NREM (Wilcoxon signed rank test, from left to right p=1.7×10^{-8}, n=56; 6.5×10^{-27}, n=229; 0.55, n=41; 4.2×10^{-17}, n=114). **G,** Fraction of non-responsive trials in NREM sleep according to sound category (Kruskal-Wallis test, p=0.11). **H,** Fraction of non-responsive trials according to sound intensity (low 50-60 dB SPL, high 70-80 dB SPL, Mann-Whitney U test, p=0.74, n=22 low, 25 high). **I,** Fraction of non-responsive trials in NREM sleep in the first and second half of the recording sessions (Wilcoxon signed rank test, p=2.2×10^{-3}, n=24 recordings). **J,** Fraction of non-responsive trials in NREM sleep when mice are head-fixed on a wheel (locomotion possible) or in a small box (no locomotion) (Mann-Whitney U test, p=1.8×10^{-1}, n=12 and 13 recordings).

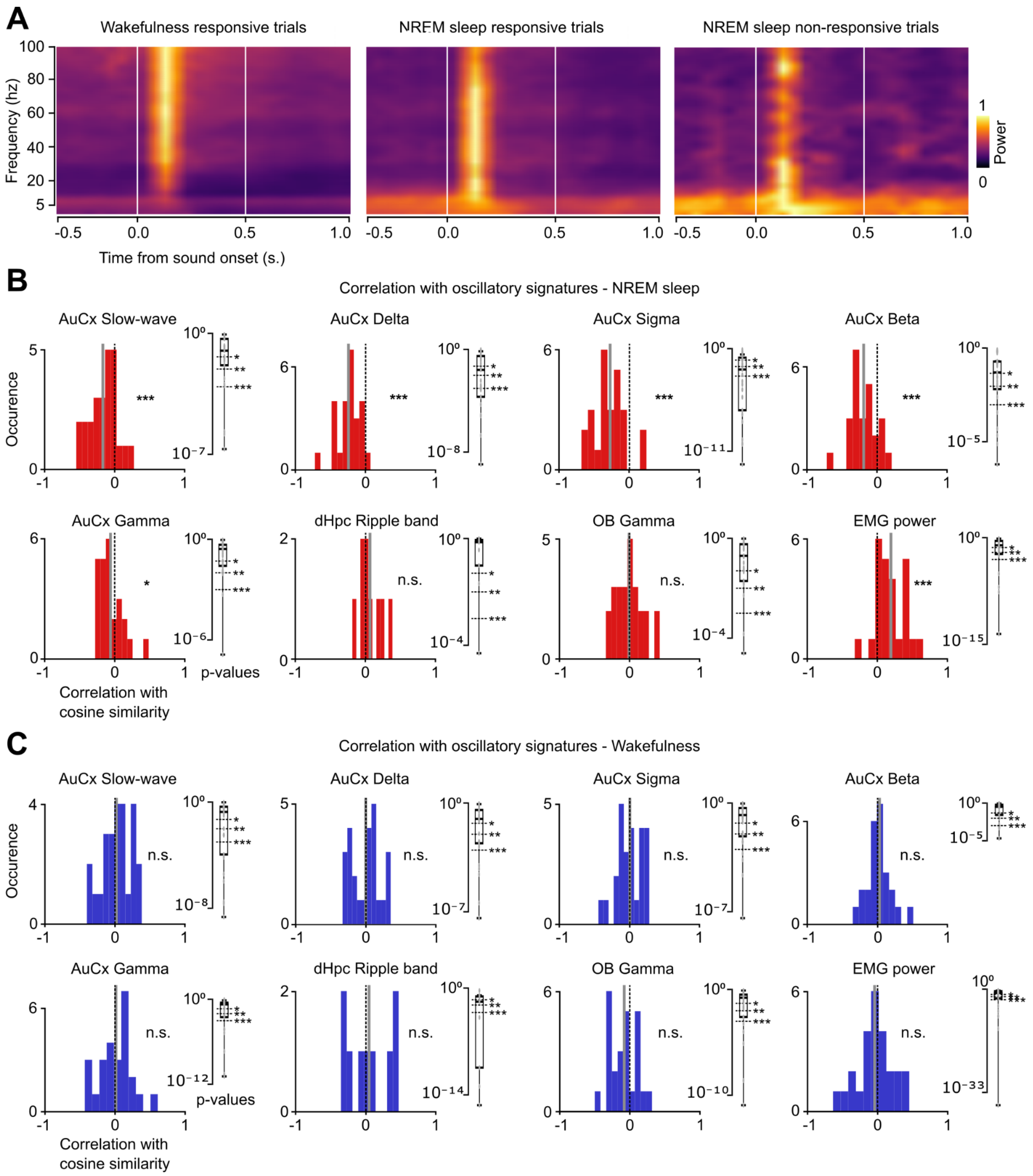

Figure S8: Population gating events are correlated to brain activity. A, Averaged spectrograms of contralateral auditory cortex LFP around sound presentation in "responsive" events in wakefulness, "responsive" and "non-responsive" events in NREM sleep. Each frequency band was divided by its maximum value from the three spectrograms to witness changes in time and allow comparison between conditions. **B,** Distribution of correlations coefficients between response similarity level and bandpower in the contralateral AuCx LFP in NREM sleep (Wilcoxon signed rank, Slow-wave: $p=2.9x10^{-4}$; Delta: $p=4.2x10^{-7}$; Sigma: $p=1.8x10^{-5}$; Beta ; $p=3.2x10^{-5}$; Gamma: $p=3.2x10^{-2}$, n=25 recordings), the ripple bandpower in the dorsal Hippocampus (Wilcoxon signed rank test, $p=5.7x10^{-1}$, n=8 recordings - only 2 mice with clear ripples were included in the analysis), and the OB gamma band and filtered EMG power (Wilcoxon signed rank test, OB Gamma: $p=8.1x10^{-1}$; EMG: $p=4.5x10^{-5}$, n=25 recordings). Pearson correlation p-values for single recordings are shown on the right. **C,** Same as **B** in wakefulness

(Wilcoxon signed rank test, AuCx SW: p=6.0x10^{-1}; AuCx Delta: p=9.8x10^{-1}; AuCx Sigma: p=9.4x10^{-1}; AuCx Beta: p=4.7x10^{-1}; AuCx Gamma: p=7.5x10^{-1} n=25 recordings; dHpc ripple band: p=7.3x10^{-1}, n=8 recordings; OB Gamma: p=8.5x10^{-2}; EMG power: p=4.9x10^{-1}, n=25 recordings)

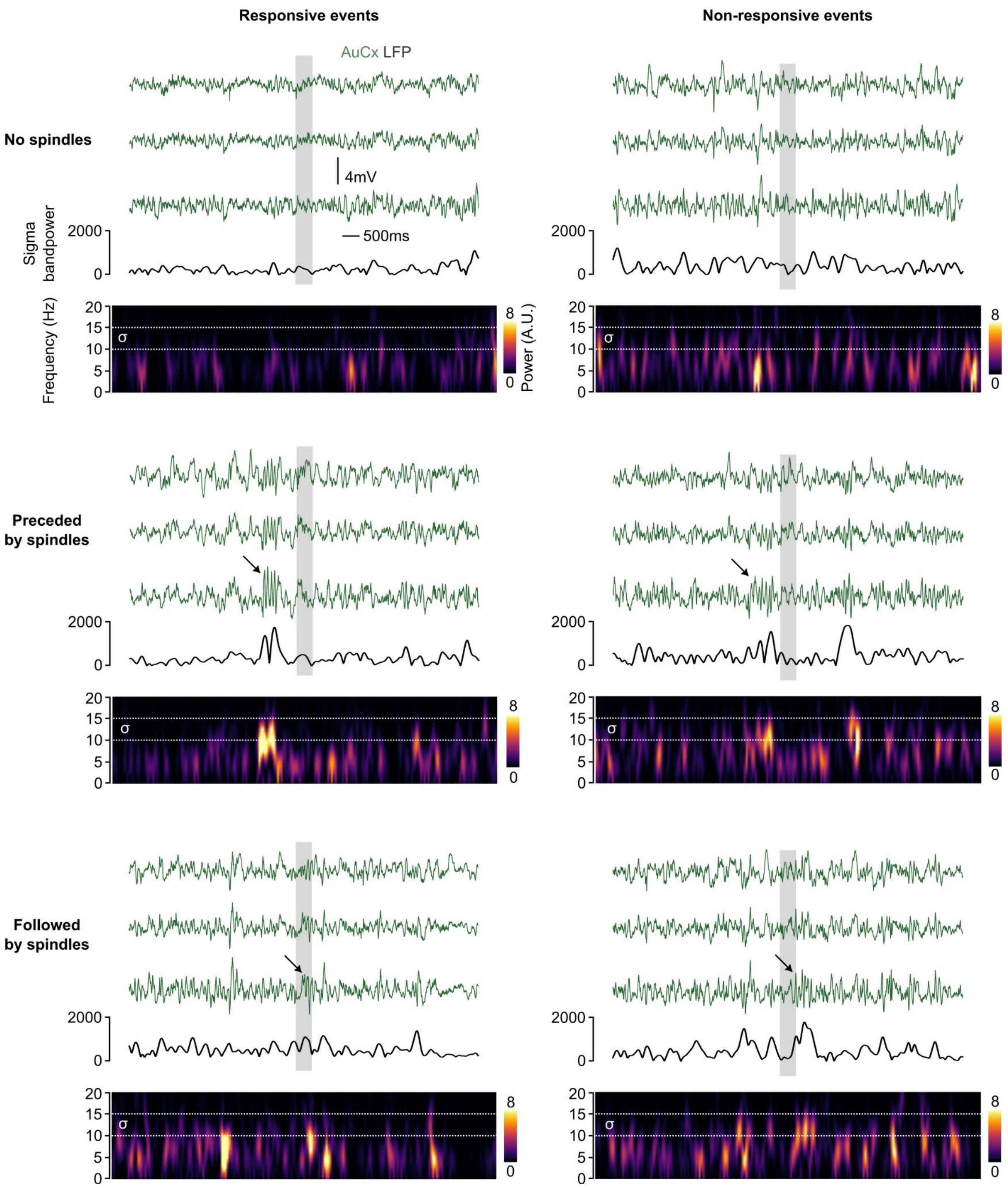

Figure S9: Responsiveness of neuronal population to sounds is not systematically linked to the occurrence of sleep spindles. LFP recording of the contralateral auditory cortex shows that “responsive” (left) and “non-responsive” (right) events can both be surrounded or not by sleep spindles. Filtered AuCx LFP traces (0.5 - 70 Hz) from 3 different channels are shown in green. The sigma (10-15 Hz) power of the bottom trace is shown in black with its spectrogram. Arrows indicate a visually detected sleep spindle event. Sound presentation is shown as a grey area.

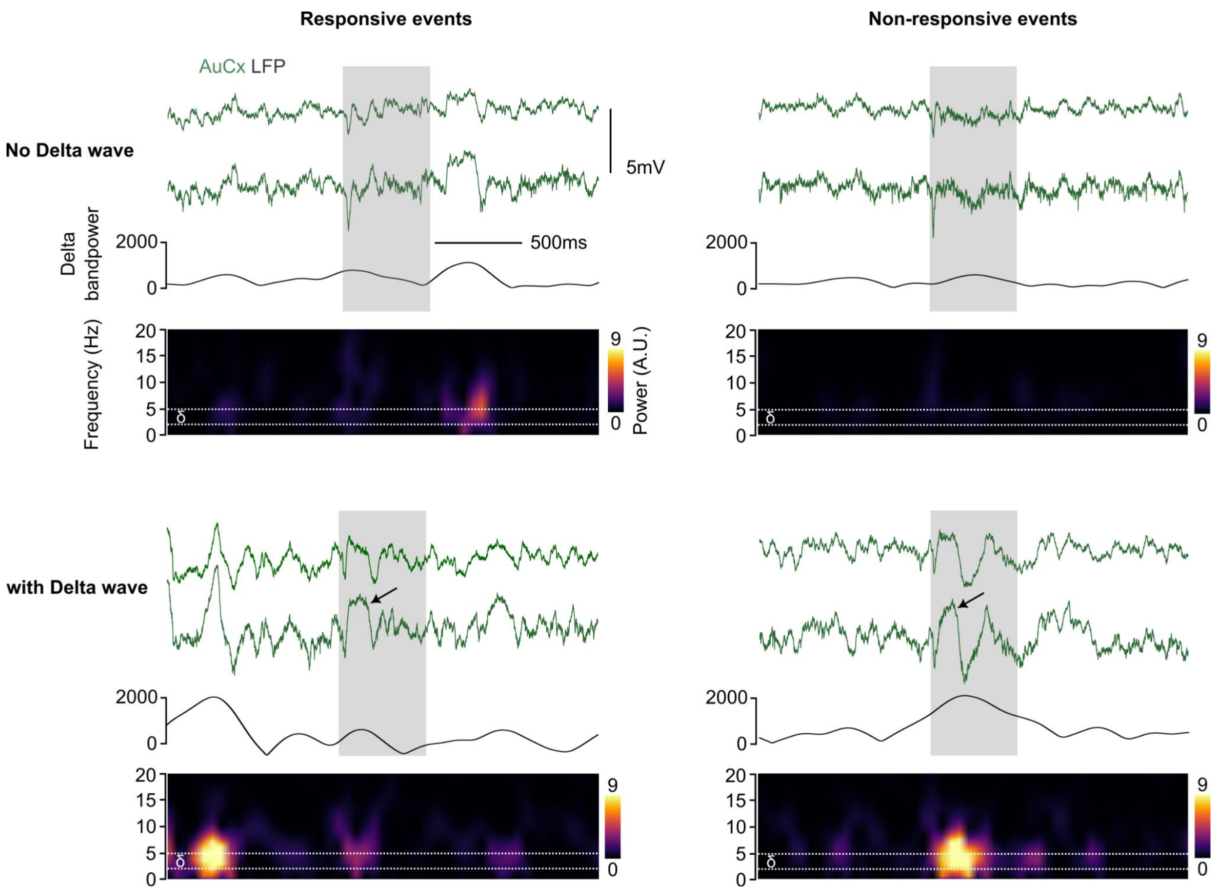

Figure S10: Responsiveness of neuronal population to sounds is not systematically linked to the occurrence of delta waves. LFP recording of the contralateral auditory cortex shows that “responsive” (left) and “non-responsive” (right) events can both co-occur or not with a delta wave in a sample recording. AuCx traces from 2 different channels are shown in green. The delta (2-5 Hz) power of the bottom trace is shown in black with its spectrogram. Arrows indicate a visually detected delta wave event during the sound presentation. Sound presentation is shown as a grey area.
